# The variability of diagnosed migraine epidemiology amongst different municipalities in southern Israel

**DOI:** 10.1186/s10194-023-01558-5

**Published:** 2023-03-13

**Authors:** Ido Peles, Mohnnad Asla, Mariya Abayev, Michal Gordon, Ali Alhoashle, Victor Novack, Rinat Ribalov, Tamar Lengil, Ron Maor, Mayera Elizur, Gal Ifergane

**Affiliations:** 1 Clinical Research Center, Soroka University Medical Center, Beer-Sheva, Israel; 2 The Faculty of Health Sciences, Ben-Gurion University of the Negev, Beer-Sheva, Israel; 3 Department of Neurology, Brain Medicine Division, Soroka University Medical Center, Beer-Sheva, Israel; 4 Medical Management, Southern District, Clalit Health Services, Tel Aviv, Israel; 5 Internal Medicine Division, Soroka University Medical Center, Beer-Sheva, Israel; 6 Teva Pharmaceutical Industries Ltd., Tel Aviv, Israel

**Keywords:** Migraine prevalence, Socioeconomic status, Variability in prevalence, Israel

## Abstract

**Background:**

Appropriate and timely diagnosis is one of the most important milestones in effective migraine care and is affected by public awareness, access to medical care, health care systems, and physicians’ knowledge. We assessed the variability in migraine diagnosis rates in different communities under universal national health coverage in Israel.

**Methods:**

In this population-based retrospective, observational, cohort study, adult (≥18 years) migraine patients were identified in the computerized database of the southern district of the Clalit Health Services Health Maintenance Organization (HMO) based on recorded diagnosis and/or purchase of specific anti-migraine acute medication (triptans). Migraine prevalence in 2018 was calculated in the entire study population and in different municipalities. We utilized a standardized (age and gender) mortality ratio (SMR) approach for comparison among the municipalities.

**Results:**

In 2018, a total of 29,938 migraine patients were identified out of 391,528 adult HMO members, with an overall prevalence (per 10,000) of migraine of 764.64 (7.65%), 1143.34 (11.43%) for women, and 374.97 (3.75%) for men. Among the municipalities, adjusted prevalence (per 10,000) ranged from 386.15 (3.86%) to 1320.60 (13.21%). The female-to-male ratio ranged from 1.8:1 to 5.1:1. Prevalence rates were positively associated with the socioeconomic status of the municipalities (Spearman rho = 0.472, *P* = 0.031).

**Conclusions:**

High variability in the prevalence of diagnosed migraine suggests underdiagnosis. Resources for awareness and educational programs should be directed to low diagnosed prevalence communities.

**Graphical Abstract:**

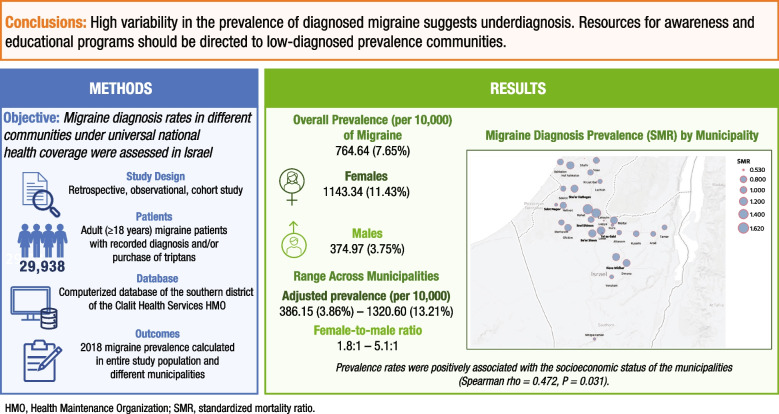

## Introduction

Migraine is a common neurological disease. The 2016 Global Burden of Diseases, Injuries, and Risk Factors (GBD) study estimated the global age-standardized prevalence of migraine to be 14.4% for adults [1], making it the most common neurological disease. A high prevalence of migraine has been repeatedly reported by surveys around the globe in different communities and ethnicities; variations have been observed, although a prevalence of < 10% has rarely been reported.

Migraine therapy, targeting both abortion of migraine attacks (acute therapy) and prevention of migraine attacks (preventive therapy) are essential for reducing suffering and disability in this common debilitating disease [2, 3]. New therapeutic options have been introduced during the last few years, enabling effective and tolerable migraine management for many patients. However, the use of any indicated acute or preventive migraine therapy starts with the diagnosis of migraine.

Appropriate and timely diagnosis is one of the most important milestones in effective migraine care and is affected by public awareness, access to medical care, health care systems, and physicians’ knowledge. Underdiagnosis of migraine is common and may be a result of patients’ beliefs and consultation patterns, health care availability, or misdiagnosis by health care providers [4].

In our day-to-day clinical practice, we have observed high variability in migraine diagnosis rates between different municipal communities in the Negev region. In this southern region of Israel, despite universal access to primary and specialist care, it seems that migraine is diagnosed in certain communities more than in others. In this study, we aimed to assess the variability in the prevalence and gender distribution of diagnosed migraine in different municipalities in southern Israel. The study focused on the municipal communities, rather than individual patients, and utilized a computerized clinical database to evaluate correlations between socioeconomic, ethnic, environmental, and health care team factors with the prevalence variability and gender distribution of migraine. The design of this research highlights the importance of considering regional, cultural, and sociodemographic factors in migraine care and suggests that resources for awareness and educational programs should be directed towards low diagnosed prevalence communities. This study could potentially be useful in designing targeted interventions to address underdiagnosis of migraine in different municipalities.

## Methods

### Clinical setup

The Israeli National Health Insurance Law requires all citizens to join 1 of 4 official non-profit health insurance organizations (HMO), which cannot legally deny membership. The Clalit Health Services (CHS), the largest Israeli HMO, divides Israel into different geographic regions; access to health services is similar for residents of each region [5]. To eliminate supply-side heterogeneity [6], we included patients residing in the southern region in our study. The Negev is the southern region of Israel, and the largest city in that region, Beer-Sheva, is considered the capital. This region includes 730,000 residents, which represents 8.2% of the Israeli population; 75% of residents are Jewish and 25% are Bedouin. In the Negev, municipal communities are ethnically homogenous. The largest health care provider in that region is the CHS, which covers approximately 67% of the region’s residents and has primary clinics available in every city, town, or settlement. The largest regional hospital in the Negev is the Soroka University Medical Center (SUMC), which is part of the hospital network for the CHS. SUMC is a tertiary 1100-bed care medical center that has > 65,000 yearly hospitalizations and approximately 200,000 yearly emergency department visits. This setup, with a single hospital serving a large region, permitted a population-based analysis, with few patients lost to follow-up and limited referral bias.

### Study population and data collection

This population-based retrospective, observational, cohort study included adults (≥18 years) with migraine in the southern district of the CHS. Patients with migraine were dentified using the CHS computerized database based on a recorded physician diagnosis (*International Classification of Diseases, Ninth Revision* [ICD-9]) of migraine (with or without aura) and/or claims for specific anti-migraine medication (triptans) at any time from 2000 to 2018. The study cohort was created in 2 steps: first, patients with physician-diagnosed migraine by a primary care provider or neurologist were identified (16,675 patients with or without triptans prescription), and second, of those without physician-diagnosed migraine, patients with a triptan prescription were identified (14,091 patients). Overall, 71.8% of patients with physician-diagnosed migraine were also prescribed triptans. Although used off-label for cluster headache treatment [7], triptans are migraine-specific [8] and are only approved in Israel for the acute treatment of migraine, which is the far more common indication. Thus, it can be assumed that the included patients with triptan prescriptions were being treated for migraine. Physician-assigned migraine diagnosis was given by either a primary care physician or neurologist. Migraine diagnoses assigned by primary care physicians were found to be highly reliable. The Landmark Study demonstrated that clinic-assigned diagnosis of migraine was validated by an expert panel based on diary data in 98% of cases [9].

The following patient demographic and clinical characteristics were collected from the central CHS computerized database: gender, age at the time of diagnosis, ethnicity, family status, education, social state score, immigrant status, comorbidities, medications, hospitalization notes, diagnostic imaging, and primary care physician visits.

Municipality characteristics were identified using data published by the Israeli Central bureau of statistics 2018 [10]. Geographic, demographic, socioeconomic, and environmental data were used for these analyses. The socioeconomic index (SEI) was used to define socioeconomic characteristics for each municipality. Each municipality was scored on the following 14 variables on a scale of 1 to 255 (1 is the lowest): average monthly income, vehicle class, new vehicle percentage, percentage of high school graduates, students, percentage of residents seeking work, percentage of residents with minimum monthly income, percentage of residents with > 2 times the average monthly income, median age, dependency ratio, percentage of families with ≥4 children, percentage of unemployment benefit recipients, percentage of income support beneficiaries, and percentage of old-age pension recipients. Based on this score, municipalities were aggregated into SEI clusters from 1 to 10 (low [1–3], medium [4–6], and high [≥7] socioeconomic status). We also used the Gini index, a measure of statistical dispersion intended to represent income inequality. The characteristics of primary care physicians practicing in CHS clinics in the different communities were provided by CHS administration.

### Statistical analysis

Data for the main variables were summarized as means and standard deviations (SDs) for normally distributed quantitative variables, medians and ranges for non-normally distributed quantitative variables, and distribution in percent for qualitative variables.

Univariate analyses were primarily used for analyses of initial personal data record datasets. For categorical variables, we used a chi-square test, with a Fisher’s exact test when needed. For continuous variables, we used a t-test for normally distributed variables, and a Mann-Whitney U-test for non-normally distributed variables.

The 2018 migraine prevalence per 10,000 adults was calculated for the overall population and each individual municipality; all calculations were made by division into the total population and female and male subgroups separately. The prevalence for each 5-year age interval was also calculated, and a standardized (age and gender) mortality ratio (SMR) approach was used for comparison among the municipalities. In addition, we calculated the female-to-male ratio of the various municipalities. These calculations were performed to examine the linear and non-linear correlation between the results of the SMR and female-to-male ratio analysis and number of variables from the Israel Central Bureau of Statistics using Pearson and Spearman tests, respectively. We also examined the correlation between the same results and a number of the physicians’ variables.

This analysis also looked at current management practices and potential gaps in the management for migraine using this study cohort. The migraine population was stratified by the source of the migraine diagnosis: physician diagnosis and/or triptan prescription. Rates of acute medication use were compared for specific (triptan [Anatomical Therapeutic Chemical (ATC) code NO2CC01–7]) and non-specific (combination pain drugs including Acamol Focus, Excedrin, Migraleve, and Rokacet Plus [ATC code NO2BE72 and NO2CX50] and opioids drugs [ATC code NO2AJ17, NO2AX02, and NO2AA55]) medications used ≥1 time during the study period for each indication.

In the last step, we performed a univariate logistic regression. The outcomes of interest were high-rate SMR (cutoff, 1.0) or high female-to-male ratio (cutoff, 3.0). The cutoff point was based on the average adjusted prevalence in the region. We have dichotomized all regional municipalities into 2 groups: high and low migraine prevalence and high and low female-to-male ratio. The model presents municipality-related characteristics associated with equal or higher than average SMR and equal or higher than average female-to-male ratio. Point estimates of association were presented by the odds ratio (OR) along with their 95% confidence intervals (95% CI).

RStudio®, version 1.4.1717 (Boston, MA), was used for these analyses. A 2-sided *P* value < 0.05 was considered statistically significant for all analyses.

### Ethics approval

Clinical investigations were conducted in accordance with the Declaration of Helsinki. The SUMC Ethics Committee (EC) provided approval for this study (reference number 0284–19). Informed consent was waived by the SMC Institutional Review Board based on the EC approval, which determined that informed consent was not needed based on the retrospective nature of the study and use of fully anonymized and deidentified patient data for analysis.

## Results

### Descriptive statistics

**Fig. 1:**
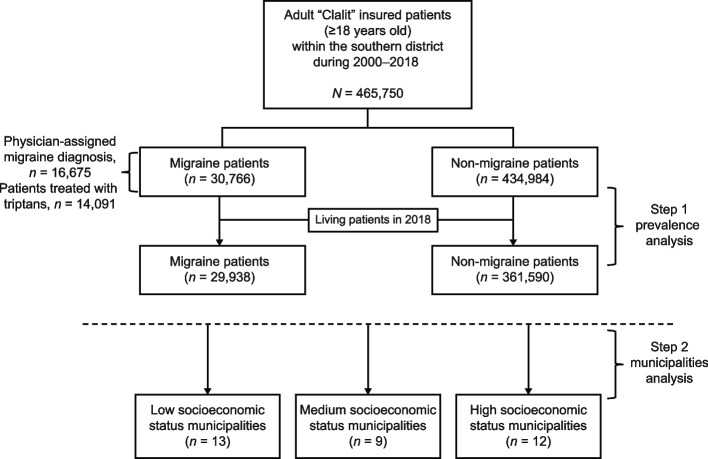
Study flow

Between 2000 and 2018, a total of 30,766 migraine patients were identified out of 465,750 adult (≥18 years old) Clalit-insured patients. Among those migraine patients, the population included 75.5% women, the mean age was 37.65 ± 14.32 years, and immigrants comprised 31.4% of the population. Most of the population was Jewish (79.9%), and the remainder was Bedouin. In 2018 alone, a total of 29,938 migraine patients were identified out of 391,528 adult HMO members, with an overall prevalence (per 10,000) of 764.64 (7.65%). The patients included in this study population came from 34 different municipalities, based on the primary care clinics to which these municipalities belong per the Clalit HMO. Less than 1% of patients did not belong to any clinic, and therefore did not enter the analysis. When the municipalities were divided according to socioeconomic status (SEI given by the Central Bureau of Statistics in Israel on a scale of 1–10), we found a total of 13 municipalities in low socioeconomic status (1–3), 9 in medium status (4–6), and 12 in high status (≥7). The study population’s flow is shown in Fig. 1.

In high socioeconomic status municipalities, patients were more likely to be Jewish (high status, 100.0%, vs medium status, 100.0%, vs low status, 30.8%; *P* < 0.001) and older (49.3 vs 47.7 vs 38.9 years; *P* < 0.001), and the population was less dense (35.3 vs 194 vs 1720 persons per square kilometer; *P* < 0.001).

In addition to the socioeconomic index, all other socioeconomic status characteristics correspond to our division. For low socioeconomic status municipalities, the natural increase rate (per 1000 persons) was higher versus medium and high-status municipalities (low, 29.3, vs medium, 18.3, vs high, 13.8; *P* < 0.001), and the average monthly salary was lower (5420 vs 8250 vs 10,300 New Israeli Shekel [NIS]; *P* < 0.001), as was the inequality index (0.35 vs 0.41 vs 0.41; *P* < 0.001). The female-to-male ratios were highest in the medium socioeconomic status municipalities and lowest in the high-status municipalities (2.9:1 vs 3.4:1 vs 2.8:1; *P* = 0.01).

Comparing physicians’ characteristics in the various municipalities, we found that in high socioeconomic status municipalities versus medium and low status municipalities, physicians were more likely to be men (high, 70.5%, vs medium, 66.1%, vs low, 63.6%; *P* = 0.955) and were older (57.0 vs 54.9 vs 50.0 years; *P* = 0.056), and there were more Jewish physicians (88.7% vs 61.7% vs 44.8%; *P* = 0.025). The percentage of physicians speaking the native language appropriate to the municipalities was lowest in the low socioeconomic status municipalities and highest in the high-status municipalities (low, 68.0%, vs medium, 68.7%, vs high, 88.7%; *P* = 0.151). The physicians included in this study had a range of experience, with the mean years of experience being 17.24 ± 10.46 years. Table 1 presents the features of the study population.

**Table 1:** Municipalities characteristics

	Low socioeconomic status (*n* = 13)	Medium socioeconomic status (*n* = 9)	High socioeconomic status (*n* = 12)	*P* value
**Demographics**				
Ethnicity: Jewish communities, n (%)	4 (30.8)	9 (100)	12 (100)	< 0.001
Female (%), mean (SD)	49.6 (1.62)	49.9 (1.13)	49.0 (1.21)	0.076
Age in 2018, mean (SD)	38.9 (5.44)	47.7 (2.42)	49.3 (3.28)	< 0.001
Total population (thousands), median [min–max]	16.3 [5.10–64.5]	24.0 [9.20–206]	7.50 [1.50–16.6]	0.003
Area (square kilometer), mean (SD)	11.3 [5.95–76.8]	117 [6.34–501]	289 [6.45–4100]	0.001
Population density (persons per square kilometer), median [min–max]	1720 [66.7–3280]	194 [26.6–3790]	35.3 [0.955–999]	< 0.001
**Socioeconomic**				
Natural increase (per 1000 persons), median [min–max]	29.3 [21.4–39.1]	18.3 [10.3–20.1]	13.8 [5.30–20.9]	< 0.001
Average monthly salary (NIS), median [min–max]	5420 [4790–7140]	8250 [6960–9110]	10,300 [7610–16,200]	< 0.001
Inequality (“Gini” index, 0 full equality), mean (SD)	0.354 (0.0283)	0.407 (0.0160)	0.413 (0.0314)	< 0.001
**Environmental**				
Recycling from waste (%), median [min–max]	4.70 [0–62.4]	15.9 [2.40–29.4]	22.4 [2.10–45.4]	0.108
Motor vehicles (per 1000 persons), median [min–max]	176 [63.8–268]	326 [276–364]	403 [276–633]	< 0.001
Forest and grove (area per square kilometer), median [min–max]	0.095 [0.06–0.33]	0.745 [0.03–3.65]	6.81 [0.07–64.8]	0.024
**Physician’s Characteristics**				
Male (%), mean (SD)	63.6 (38.2)	66.1 (28.6)	70.5 (25.3)	0.955
Native speakers appropriate (%), mean (SD)	68.0 (37.8)	68.7 (20.4)	88.7 (18.9)	0.151
Graduates of medicine schools in Israel (%), mean (SD)	24.9 (37.0)	27.9 (25.3)	46.7 (42.8)	0.366
Physician’s age, mean (SD)	50.0 (6.10)	54.9 (3.89)	57.0 (6.51)	0.056
Jews (%), mean (SD)	44.8 (41.8)	61.7 (32.3)	88.7 (18.9)	0.025
**Migraine Characteristics**				
Overall prevalence (per 10,000; %), median [min–max]	6.50 [4.10–13.2]	6.72 [3.86–9.17]	8.38 [5.20–13.2]	0.054
Standardized prevalence ratio, median [min–max]	0.800 [0.550–1.62]	0.880 [0.530–1.16]	1.04 [0.640–1.44]	0.079
Standardized female-to-male ratio, median [min–max]	2.91:1 [1.76–5.12]	3.43:1 [3.04–4.13]	2.78:1 [2.12–4.40]	0.01

*SD* standard deviation, *NIS* New Israeli Shekel

### Standardized (age and gender) migraine prevalence

**Fig. 2:**
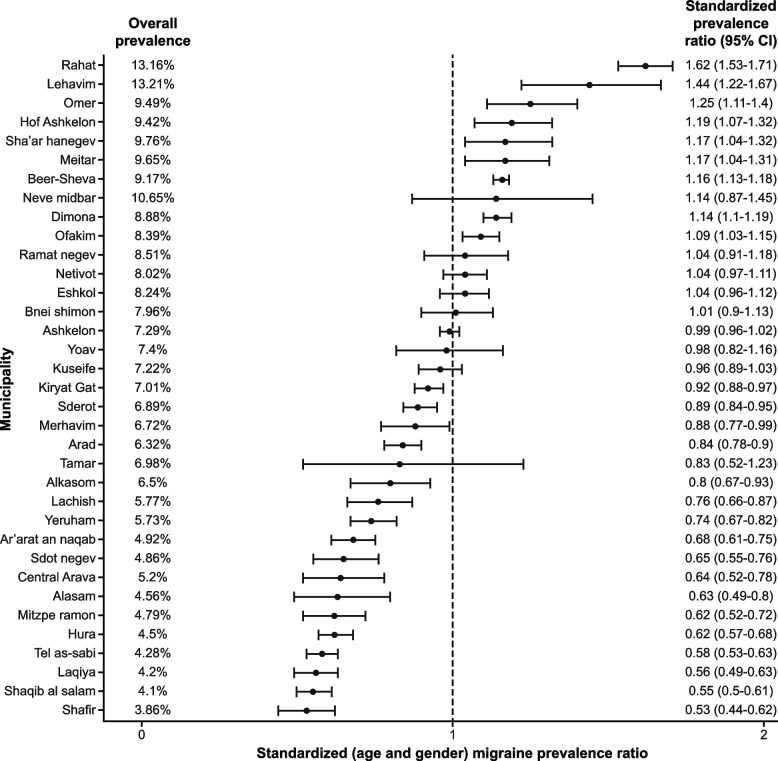
Standardized (age and gender) prevalence ratios (SMR). SMR, standardized mortality ratio; CI, confidence interval

Among the municipalities, overall prevalence ranged from 386.15 (3.86%) to 1320.60 (13.21%). Figure 2 displays the municipalities by order of SMR (standardized for age and gender); municipalities with a SMR > 1 are those in which the proportion of migraine patients observed was higher than expected. Rahat, Lehavim, and Omer are at the top of the list with SMRs ranging from 1.25 to 1.62, while several Bedouin municipalities like Laqiya, Shaqib al-Salam, and Hura, along with the Shafir municipality, are at the bottom of the list with rates < 0.65.

We assessed the geographical distribution of the age- and gender-adjusted (of the general population) migraine rates; no significant trends were observed in this analysis, including proximity to conflict areas for other diseases of a similar nature. There is a trend of higher migraine rates in Beer-Sheva, the central city of the district, where the advanced medical services are located, but this is not statistically significant.

Table 2 displays a univariable analysis of factors found to be associated with standardized (age and gender) migraine ratios (SMR). High social state score (OR, 5.33; 95% CI: 1.23–27.0), and other socioeconomic characteristics, including low natural increase < 18 per 1000 persons (OR, 4.68; 95% CI: 1.09–22.9) and high average monthly salary > 9500 NIS (OR, 17.0; 95% CI: 2.41–350), were associated with higher standardized (age and gender) migraine ratios (SMR). Municipalities’ ethnicity, population density, inequality, and physicians’ characteristics, including age, gender, and ethnicity, were not significant risk factors for the dependent variable.

**Table 2:** Standardized (age and gender) migraine prevalence ratios (SMR)

	Pearson rank	Spearman rank	OR^a^	CI		*P* value
				Lower	Upper	
**Demographics**						
Jewish communities (ethnicity)	0.213	0.296	3.23	0.63	24.7	0.19
High population density > 200 (persons per square kilometer)	0.122	0.080	1.25	0.31	5.19	0.75
**Socioeconomic**						
High social state score (“Eshkol” index > 6)	0.419	0.472	5.33	1.23	27.0	0.031
Low natural increase < 18 (per 1000 persons)	0.471	0.563	4.68	1.09	22.9	0.044
High average monthly salary > 9500 (NIS)	0.458	0.447	17.0	2.41	350	0.015
High inequality (Gini index > 0.4)	0.352	0.462	2.83	0.69	12.8	0.16
**Physician’s Characteristics**						
Mean age > 50 years	0.362	0.360	1.85	0.30	15.1	0.52
Male > 35%	0.028	0.025	5.42	0.73	112	0.15
Native speakers appropriate > 50%	0.029	0.045	1.29	0.18	11.0	0.80
Graduates of medicine schools in Israel > 25%	0.071	0.026	1.12	0.27	4.73	0.87
Jews > 75%	0.331	0.386	2.57	0.61	11.8	0.20

*SMR* standardized mortality ratio, *OR* odds ratio, *CI* confidence interval, *NIS* New Israeli Shekel

^a^Uni-variable logistic regression model results; the outcome is high-rate SMR (cutoff, 1.0). It presents municipality-related characteristics associated with equal or higher than average SMR

### Standardized (age) female-to-male ratios

**Fig. 3:**
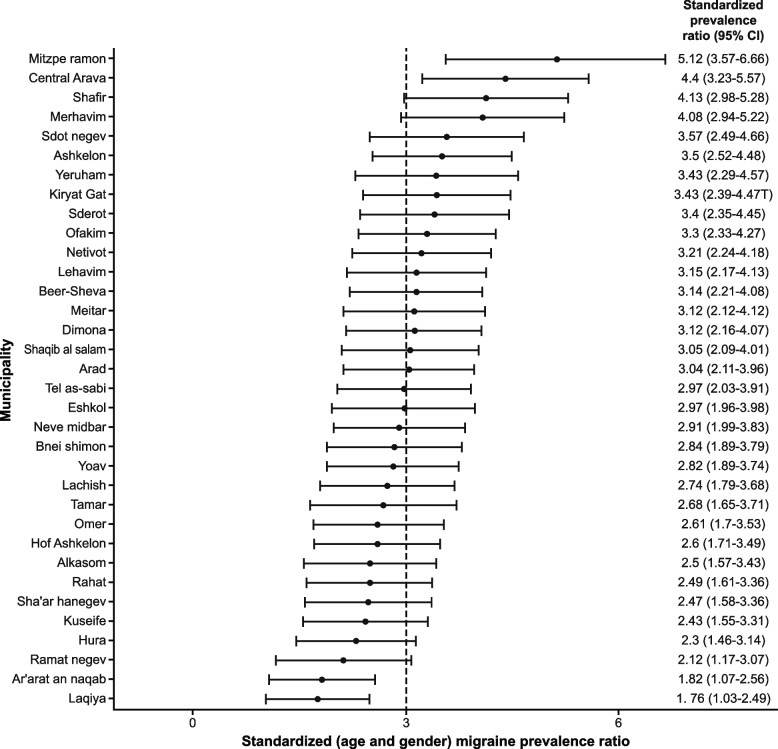
Age-adjusted female-to-male ratios. CI, confidence interval

Figure 3 presents the municipalities by order of the age-adjusted (of the general population) female-to-male ratios. The female-to-male ratio ranged from 1.76:1 to 5.12:1. Mitzpe Ramon (5.12:1), Central Arava (4.4:1) and Shafir (4.13:1) are at the top of the list, while a number of Bedouin municipalities like Laqiya (1.76:1), Ar’arat an Naqab (1.82:1), and Hura (2.3:1), along with the Ramat Negev municipality (2.12:1), are at the bottom of the list.

Table 3 displays a univariable analysis of factors found to be associated with adjusted-to-age, female-to-male ratios. The female-to-male ratios were higher in the medium and high socioeconomic status municipalities compared to low-status municipalities (OR, 2.13; 95% CI: 0.53–9.27). Ethnicity was the factor most associated with higher female-to-male ratios; largely Jewish municipalities had higher ratios (OR, 14.2; 95% CI: 2.12–287). Physicians’ characteristics, direct, and other socioeconomic characteristics, other than population density, did not pose a significant risk factor for the dependent variable.

**Table 3:** Standardized (age) female-to-male ratio

	Pearson rank	Spearman rank	OR^*^	CI^*^		*P* value^*^
				Lower	Upper	
**Demographics**						
Jewish communities (ethnicity)	0.494	0.547	14.2	2.12	287	0.020
High population density > 55 (persons per square kilometer)	0.122	0.010	5.44	1.04	42.5	0.026
**Socioeconomic**						
High social state score (“Eshkol” index > 3)	0.149	0.113	2.13	0.53	9.27	0.29
High natural increase > 18 (per 1000 persons)	0.179	0.064	1.67	0.41	7.08	0.48
Low average monthly salary < 9500 (NIS)	0.031	0.108	4.20	0.78	33.0	0.12
High employee inequality (Gini index > 0.4)	0.283	0.227	2.78	0.68	12.3	0.16
**Physician’s Characteristics**						
Mean age, > 55 years	0.339	0.252	2.62	0.59	13.0	0.21
Male > 35%	0.261	0.150	6.36	0.86	132	0.11
Native speakers appropriate > 80%	0.016	0.102	1.47	0.36	6.24	0.59
Graduates of medicine schools in Israel < 50%	0.118	0.006	2.95	0.52	23.6	0.24
Jews > 50%	0.247	0.119	1.25	0.26	6.28	0.78

*SMR* standardized mortality ratio, *OR* odds ratio, *CI* confidence interval, *NIS* New Israeli Shekel

^*^Uni-variable logistic regression model result; the outcome is high-rate female-to-male ratio (cutoff, 3.0). It presents municipality-related characteristics associated with equal or higher than average female-to-male ratio

## Discussion

In this study, we evaluated the variability of the prevalence and gender distribution of diagnosed migraine in different municipalities in southern Israel, using the computerized database of a large HMO. The overall prevalence was found to be 7.65% with a female-to-male ratio of 3:1, yet both parameters were highly variable with the age- and gender-adjusted prevalence ranging from 3.86% to 13.21% and the female-to-male ratio ranging from 1.76:1 to 5.12:1.

Unlike previous epidemiological studies, which focused on migraine patients, our study focuses on municipal communities. We evaluated correlations between the characteristics of the community and the primary physicians serving that community with the prevalence and gender distribution of migraine.

The epidemiology of migraine is poorly reflected by clinical databases since many patients are not diagnosed for various reasons. Epidemiological studies have therefore focused on community surveys, which have been conducted in various settings worldwide. The 2016 GBD study estimated the prevalence of migraine in adults to be 14.4% worldwide [1], while the female-to-male ratio is typically 3:1 [11]. The prevalence of diagnosed migraine in our study is lower and probably does not reflect the true prevalence of migraine in this population in southern Israel, only the fraction that consulted their physicians and were diagnosed. It is therefore reasonable to assume that the high variability of diagnosed migraine reflects variable degrees of underdiagnosis, not necessarily actual prevalence.

We found that a low prevalence of diagnosed migraine was associated with lower socioeconomic status of the municipality, while gender distribution was associated with ethnic variations between the municipalities (lower female-to-male ratios in Arab municipalities). The characteristics of the physicians in the local primary clinics (gender, ethnicities, experience, or training) were not associated with either factor (prevalence or gender distribution).

The prevalence of migraine has repeatedly been found in the literature to be higher in individuals with lower income [12–14]. Our findings demonstrated a lower prevalence of diagnosed migraine in low socioeconomic status communities. This discrepancy may suggest that the underdiagnosis of migraine in poor communities is even greater than reflected by the presented variability. Sociodemographic factors have previously been reported to influence migraine diagnosis and care, but the lack of medical insurance was considered the major barrier to care [15]. Our findings suggest that even when access to medical care is universal, such factors present a barrier to diagnosis.

The variability in the gender distribution of migraine was high, yet not easy to interpret. Low female-to-male ratios were associated with Bedouin communities, but not with socioeconomic status. Gender distribution was not associated with the natural population growth, making multiple pregnancies an unlikely cause for underdiagnosis. Community surveys preformed in Muslim countries (Pakistan and Saudi Arabia [16–18]) have previously reported a relatively low female-to-male ratio (1.5:1) among migraine patients, as compared with western European and US surveys. It is difficult, though, to determine if this different gender pattern represents a different gender distribution due to lower migraine burden among Muslim women or possible diagnosis bias related to cultural barriers. Our results display the same pattern. Further research is needed to elucidate this point.

Barriers to migraine diagnosis could be related to patients’ awareness, knowledge, and beliefs, which affect headache consultation patterns; access (financial or geographical) to medical care; quality of patient-physician communication; and physicians’ attitudes, beliefs, and knowledge in headache care. Since migraine diagnosis is established based on subjectively described symptoms, we postulated that physicians who share the language and culture of the community they are serving may be more likely to better diagnose patients with migraine. We tried to examine this hypothesis by evaluating the characteristics of the primary care physicians serving each community. This hypothesis was not supported by our findings: the age, gender, experience, and training of the physicians in the various municipalities were not associated with diagnosed migraine prevalence, nor was the proportion of physicians fluent in the municipality’s spoken language (Hebrew/Arabic).

The major weakness of our study is that it relies on physician diagnosis of migraine, and therefore cannot indicate the true prevalence of migraine and other headache disorders. This limits the generalizability of the study findings only to patients with migraine who sought care. In addition, electronic medical records (EMR) fail to capture migraine activity or severity. Another limitation of our study is that not all of the physicians have experience in the field of migraine, and therefore, it is possible that not all patients included in this study population were correctly diagnosed. Given that the current results support the underdiagnosis of migraine in the study population, increasing the specificity of diagnosis would likely only strengthen that finding.

Finally, the retrospective design of our study carries the inherit limitation of working with an established dataset, which is prone to reporting bias and may have lower data quality compared to data collected prospectively; this can potentially result in misclassification of study participants. Its major strength is capturing diagnosis rates in a large population residing in a diverse cluster of relatively homogenous communities, with similar access to care.

One of the most significant barriers to migraine care is diagnosis [4]. Major efforts worldwide are focused on headache-related medical education, in the hope that improving physicians’ skills and knowledge in the headache field will increase migraine diagnosis rates. Our findings suggests that there is need for more community-focused disease awareness campaigns in line with those launched in recent years [19]. Many campaigns address communities with higher education levels and financial resources. They often ignore the digital divide and do not address weakened communities, increasing inequity instead of reducing it. Our findings suggest that public education and disease awareness efforts that focus on low socioeconomic status communities may have a larger impact on headache burden. Monitoring migraine diagnosis rates from EMR systems can help focus campaigns to vulnerable populations. Interventional studies are needed to support this approach and provide recommendations for different types of campaigns in different populations.

## Data Availability

The data that support the findings of this study are available from the corresponding author upon reasonable request.

## Abbreviations

ATC: Anatomical Therapeutic Chemical
CHS: Clalit Health Services
CI: Confidence interval
EC: Ethics committee
EMR: Electronic medical records
GBD: 2016 Global Burden of Diseases, Injuries, and Risk Factors
HMO: Health maintenance organization
ICD-9: International Classification of Diseases, Ninth Revision
NIS: New Israeli Shekel
OR: Odds ratio
SD: Standard deviation
SEI: Socioeconomic index
SMR: Standardized mortality ratio
SUMC: Soroka University Medical Center
